# Alpinetin Suppresses Effects of TGF-β1 on Stimulating the Production and Organization of Fibrotic Markers in Human Primary Dermal Fibroblasts

**DOI:** 10.3390/cells11172731

**Published:** 2022-09-01

**Authors:** Nitwara Wikan, Saranyapin Potikanond, Wutigri Nimlamool

**Affiliations:** Department of Pharmacology, Faculty of Medicine, Chiang Mai University, Chiang Mai 50200, Thailand

**Keywords:** skin fibrosis, dermal fibroblasts, TGF-β1, myofibroblasts, alpinetin, mesenchymal features

## Abstract

Overgrowths of dermal fibroblasts and myofibroblast phenoconversion in response to TGF-β stimulation are the hallmarks of skin fibrosis. Constitutive activation of dermal fibroblasts by TGF-β induces the excessive production of extracellular matrix as well as certain key intracellular proteins which form a complex interaction network. Current therapies include monoclonal anti-bodies against TGF-β and surgery, but these treatments generally elicit a limited effect on certain kinds of skin fibrosis. In the current study, we investigated the effects of alpinetin (AP) on human primary dermal fibroblasts (HPDFs) stimulated with TGF-β1. Results demonstrated that AP exhibited strong inhibitory effects on TGF-β1-induced proliferation and migration of HPDFs. AP also inhibited TGF-β1-induced morphological changes of fibroblasts to myofibroblasts, and these were found to be from its effects on blocking actin stress fiber formation and organization. The expression of major fibrotic molecules including α-SMA and type I collagen upon TGF-β1 stimulation was also inhibited by AP. In addition, AP attenuated TGF-β1-induced production and organization of vimentin, β-catenin, and N-cadherin, important for the pathophysiology of skin fibrosis. In conclusion, we revealed that AP has an ability to reverse the fibrotic effects of TGF-β1 at the cellular level, and this discovery suggests the therapeutic potential of AP for skin fibrosis.

## 1. Introduction

When skin is damaged, the body immediately initiates the complex series for repairing a wound. Although some skin abrasions can be repaired so perfectly that the site is left scarless, this ideal outcome of wound healing rarely occurs because any break in cutaneous integrity including burn, injury, deep trauma, and surgery can result in hypertrophic scars [1,2]. Hyperproliferation of dermal fibroblasts and overproduction of extracellular matrix (ECM) are major characteristics of skin fibrosis [3], and an overly aggressive healing process may result in keloid scars which are difficult to treat [4].

Abundant experimental evidence has proved that TGF-β drives tissue fibrosis through direct activation of its TGF-β receptors [5,6]. Likewise, this growth factor is shown to be the most critical regulatory factor that stimulates the activation of pathological scar fibroblasts [7,8,9]. TGF-β stimulates phenoconversion from fibroblasts to myofibroblasts which display mesenchymal features, such as a dramatic production of vimentin intermediate filaments [10]. Another distinct feature of myofibroblasts upon TGF-β induction is the organization of contractile fibers composed of a rich network of actin–myosin bundles and the high expression of α-smooth muscle actin (α-SMA), which is a key marker of mature myofibroblasts [11,12]. In addition, activated myofibroblasts in fibrotic tissues express and deposit high levels of structural collagens along with several different extracellular matrix components [13]. It has been demonstrated that TGF-β enhances the production of type I and type III collagen yet inhibits collagenase activity, resulting in continuous growth of keloids [14,15]. Additionally, TGF-β could strongly regulate β-catenin expression in fibroblasts from different tissues [16,17], and this protein controls cell motility and epithelial–mesenchymal transition [18,19]. In particular, sustained activity of β-catenin in dermal fibroblasts promotes fibrosis by upregulating expression of extracellular matrix protein-coding genes [20], and β-catenin has been demonstrated to play a regulatory role in keloid fibroblast proliferation and apoptosis [21]. Human fibroblasts have been proven to express N-cadherin [22], and TGF-β can induce the expression of N-cadherin, which is involved in myofibroblast invasion and migration [23]. Specifically, pathologic proteolytic processing of N-cadherin has been identified as a marker of human fibrotic diseases [24]. Since TGF-β has diverse fibrotic effects, this growth factor has become a potential therapeutic target for skin fibrotic diseases.

Alpinetin (AP), a plant flavonoid predominant in various plants, has been shown to exhibit many pharmacological effects [25,26,27,28]. One study reported the protective effect of this flavonoid on chronic obstructive pulmonary disease [29]. This study provided interesting results, showing that AP reduces the expression of TGF-β1 and α-SMA and concluding that AP inhibits apoptosis, inflammation, and fibrosis of alveolus pulmonis cells in rat models. However, the potential anti-fibrosis activities of AP in human dermal fibroblasts (HPDFs) have not been explored. Therefore, in this study we focused on investigating the inhibitory effects of AP on TGF-β-induced proliferation and myofibroblast phenoconversion of human primary dermal fibroblasts. We defined the possible mechanisms of AP on reversing the fibrotic potency of TGF-β, and our data are convincing that AP may have therapeutic value in treating skin fibrotic diseases.

## 2. Materials and Methods

### 2.1. Cell Culture

Human primary dermal fibroblasts (HPDFs) (PCS-201-010™) were purchased from ATCC (Manassas, VA, USA). This primary dermal fibroblast cell was originally isolated from foreskin of a male neonatal donor. Cells were cultured in fibroblast complete growth media (Fibroblast Basal Medium (ATCC PCS-201-030) supplemented with Fibroblast Growth Kit–Low Serum (ATCC PCS-201-041)) (ATCC, Manassas, VA, USA). HPDFs were cultured in the culture flasks placed in the incubator at 37 °C, 5% CO_2_ atmosphere. When the cells had reached approximately 80% confluence and were actively proliferating, they were passaged. Human primary dermal fibroblasts used in this study were maintained to be at low passage (passage 1–5) which was not allowed to exceed passage 5. Experiments were performed in at least three individual replicates.

### 2.2. Cell Viability Assay

On the basis that we mainly focused on defining the molecular mechanism of action at the signaling level underlying antifibrotic action of alpinetin (AP), the utilized compound must have high purity to rule out the possible confounding actions of contaminants. Therefore, alpinetin (AP) (7-Hydroxy-5-methoxyflavanone) was commercially obtained from Chengdu Biopurify Phytochemicals Ltd., (Chengdu, China). The purity of AP was certified by the company to be 99.33% by HPLC-DAD analysis. The metabolic effect of AP was determined by MTT assay. HPDFs at 5 × 10^4^ cells were seeded into 96-well plates (3599, Corning, Kennebunk, ME, USA) overnight. Cells were left untreated or treated with varied concentrations of AP (prepared by a 2-fold dilution in FBS-free media, with maximum concentration of 800 µM). For testing the suppressing effect of AP on TGF-β1-stimulated growth of HPDFs, similar preparation of AP was prepared in the media with the presence of 10 ng/mL of TGF-β1. The cells were treated for 24 h, 48 h, and 72 h in the incubator at 37 °C, 5% CO_2_ atmosphere. After each time point, media were replaced with new FBS-free media containing 0.5 mg/mL of MTT reagent, and cells were incubated for 2 h at 37 °C, 5% CO_2_ atmosphere. The MTT solution was discarded, and cells were washed with FBS-free media 1 time before adding 100 µL of 100% DMSO into each well to dissolve the purple formazan complex. Measurement was conducted at 570 nm by a plate reader (BioTek Instruments, Winuski, VT, USA).

### 2.3. Cell Proliferation Assay

HPDFs were seeded at a very low density (0.5 × 10^5^ cells/mL) (125 cells/mm^2^) in 24-well plates in 500 µL of complete media to create a homogeneous distribution of single HPDFs for 24 h. The cells were treated with 50 µM AP in serum-free media, and the images of cell proliferation were captured (with 10× magnification) at 0, 24, 48, and 72 h by an Axio Vert.A1 microscope (Carl Zeiss, Jena, Germany). Additionally, the total number of cells at each time points were counted by using the CellDrop™ Automated Cell Counter (DeNovix Inc., Wilmington, DE, USA).

### 2.4. Cell Migration Assay

HPDFs were seeded and cultured in complete media in 24-well plates until they occupied the entire surface of the dish. A scratched wound was created by a sterile 200 µL pipette tip. Then, culture supernatants were discarded, and cells were washed with sterile PBS for 3 times. The well was refilled with serum-free media (SFM) containing 10 ng/mL of TGF-β1 or SFM containing 10 ng/mL of TGF-β1 with 50 µM of AP. The scratch was observed at 0, 24, 48, and 72 h by an Axio Vert.A1 microscope.

### 2.5. Immunofluorescence Microscopy

HPDFs (1.5 × 10^5^ cells) were seeded on 12 mm diameter cover glasses (Thermo Fisher Scientific, Braunschweig, Germany) in 24-well plates (3524, Corning, Kennebunk, ME, USA) overnight. Then, cells were left untreated, treated with 10 ng/mL of TGF, or 10 ng/mL of TGF with 50 µM of AP for 48 h. Cells were fixed with 4% formaldehyde solution for 15 min at room temperature (RT). After washing with PBS 3 times, cells were permeabilized with 0.3% TrironX-100 for 5 min followed by staining with appropriate antibodies overnight at 4 °C. Primary antibodies (all from Cell Signaling Technology (Boston, MA, USA)) included a rabbit anti-nuclear Ki-67 protein antibody (catalog number 9129), a mouse anti-α-Tubulin antibody (DM1A) (catalog number 3873), a rabbit anti-vimentin antibody (D21H3) (catalog number 5741), a rabbit anti-β-catenin antibody (D10A8) (catalog number 8480), a rabbit anti-N-cadherin antibody (D4R1H) (catalog number 13,116), a mouse anti-α-SMA antibody (1A4) (catalog number 48,938), and a rabbit anti-Col1A1 antibody (catalog number 84,336).

After three washes, cells were stained with appropriate secondary antibodies including Alexa488-conjugated goat anti-rabbit IgG or Alexa488-conjugated goat anti-mouse IgG antibodies (Thermo Fisher Scientific, USA) and DAPI for 2 h, at RT, followed by three washes. In some experiments, filamentous actin (F-actin) was stained with DyLight-594-Phalloidin (catalog number12877) (Cell Signaling Technology, Boston, MA, USA).

Cover glasses were mounted with 10 µL of anti-fade mounting media and observed under a fluorescent microscope (Axio Vert.A1) (Carl Zeiss, Jena, Germany).

### 2.6. Western Blot Analysis

Cell lysates were prepared by adding 150 µL of Laemmli buffer (reducing buffer) into each well of 24-well plates. Lysate samples were collected into vial tubes and heated at 95 °C for 5 min. Cell lysates were separated by SDS-PAGE gel electrophoresis and transferred to PVDF membrane. After blocking with 5% BSA/TBST at RT for 1 h, the immunoblots were probed with appropriate primary antibodies (all from Cell Signaling Technology (Boston, MA, USA)) against N-cadherin (D4R1H) (catalog number 13,116), β-catenin (D10A8) (catalog number 8480), vimentin (D21H3) (catalog number 5741), α-SMA (1A4) (catalog number 48,938), Col1A1 (catalog number 84,336), and β-actin (8H10D10) (catalog number 3700) followed by appropriate secondary antibodies which included an anti-mouse IgG conjugated with IRDye^®^800CW (catalog number 926-32210) and an anti-rabbit IgG conjugated with IRDye^®^680RT (catalog number 926-68071). Fluorescent signal was detected with the Odyssey^®^ CLx Imaging System (LI−COR Biosciences, Lincoln, NE, USA), and densitometric analysis was performed by the ImageJ software (version 1.51j8) (NIH, Bethesda, MD, USA).

### 2.7. Statistical Analysis

Data are presented as mean ± standard error of mean (SD). Differences between groups were analyzed by one-way or two-way analysis of variance (ANOVA) (with Tukey’s post hoc multiple comparisons on RAW data reads) or an unpaired T-TEST. Significant differences compared with appropriate controls are denoted with asterisks, * *p* < 0.05. All experiments were repeated at least three times independently.

## 3. Results

### 3.1. Alpinetin (AP) Inhibits TGF-β1-Induced Proliferation and Migration of Human Primary Dermal Fibroblasts (HPDFs)

**Figure 1:**
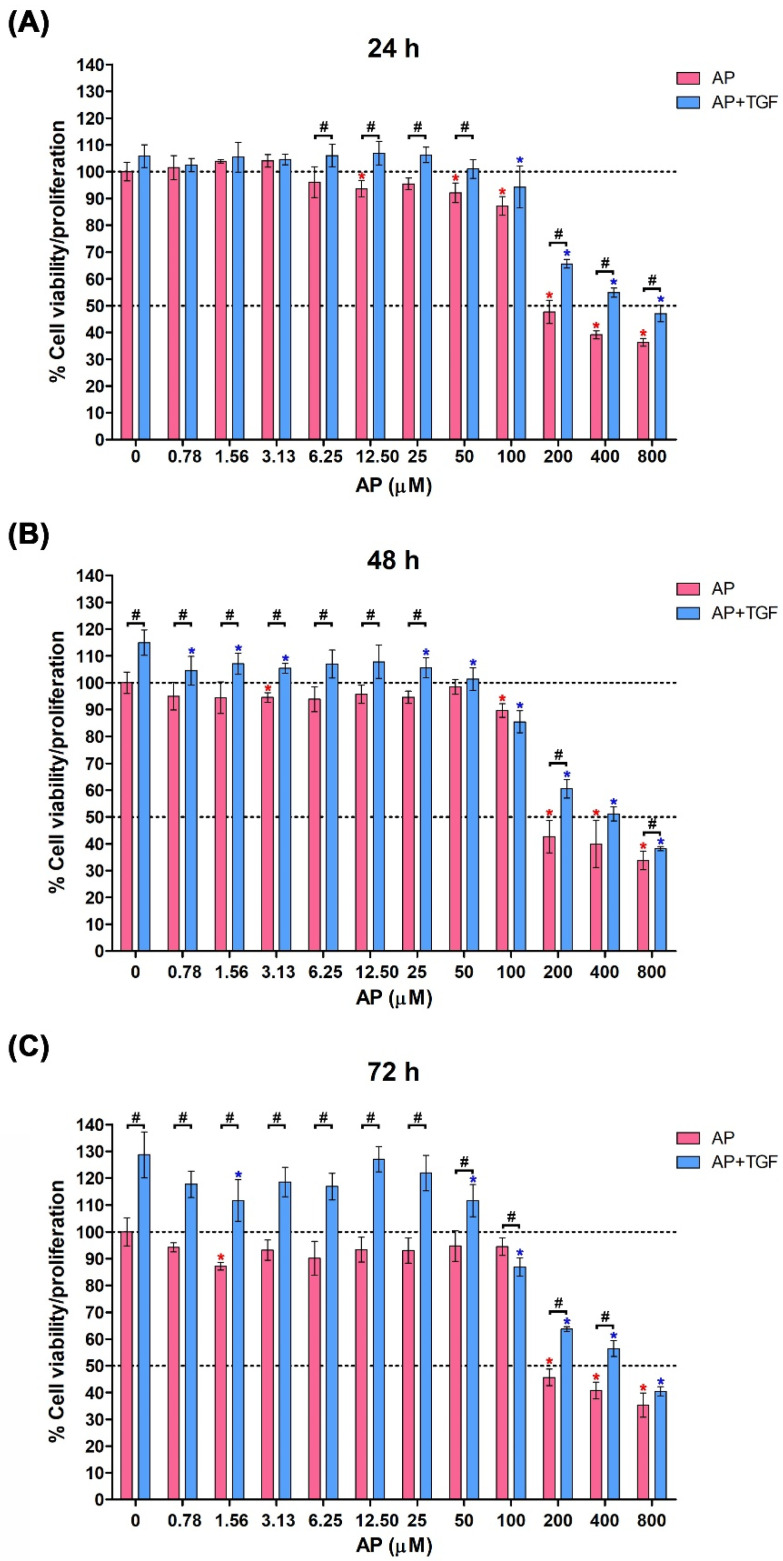
Alpinetin (AP) inhibits proliferative responses of human primary dermal fibroblasts (HPDFs) to TGF-β1. Cell viability assay of HPDFs treated with AP (0–800 µM) with or without the presence of 10 ng/mL TGF-β1 at 24 h (**A**), 48 h (**B**), and 72 h (**C**). Data are averages from three independent experiments and represented as mean ± SD of three independent experiments; ^#^ *p* < 0.05 or * *p* < 0.05 (red asterisk (compared to the untreated group), and blue asterisk (compared to TGF-β1-treated group)).

We first determined the effect of TGF-β1 and AP on cell viability and proliferation of HPDFs by MTT essay. Data showed that the cell viability of HPDFs treated with AP at concentrations up to 50 μM was maintained more than 90% over the course of 72 h (Figure 1A–C). However, AP at concentrations above 100 μM caused a decrease in cell viability/proliferation with IC50 value being around 200 μM (Figure 1A–C). Hence, 50 μM was selected as an optimal concentration of AP to be applied for all experiments. When HPDFs were exposed to TGF-β1 (10 ng/mL), the percent cell viability/proliferation significantly increased in a time-dependent manner, which was approximately 120% (at 72 h) compared to that of the control group (100%) (Figure 1C). These proliferative effects of TGF-β1 were suppressed by AP in a concentration-dependent fashion, and the IC50 value of AP was approximately 400 μM (Figure 1A–C). These data led us to hypothesize that AP may inhibit proliferative effects of TGF-β1 on human dermal fibroblasts.

**Figure 2:**
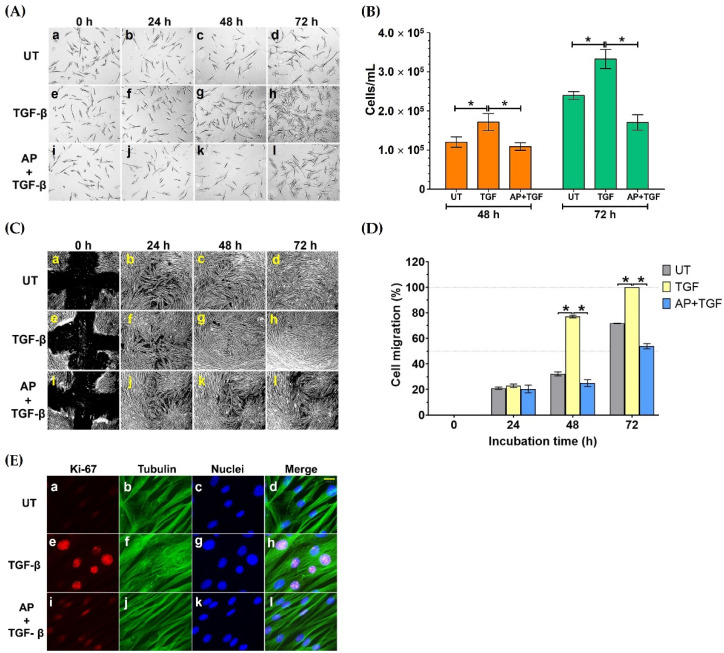
AP suppresses TGF-β1-induced proliferation and migration of HPDFs. (**A**) Colony formation of HPDFs (untreated (UT), treated with 10 ng/mL of TGF-β1, or treated with 10 ng/mL of TGF-β1 with 50 μM of AP) at 0 h, 24 h, 48 h, and 72 h. Figure A(**a**–**d**) present the untreated cells at 0 h, 24 h, 48 h, and 72 h, respectively. Figure A(**e**–**h**) present TGF-β1-treated cells at 0 h, 24 h, 48 h, and 72 h, respectively. Figure A(**i**–**l**) present cells treated with TGF-β1 and 50 μM of AP at 0 h, 24 h, 48 h, and 72 h, respectively. (**B**) Number of HPDFs (untreated, TGF-β1-treated, or TGF-β1-treated with the presence of 50 μM of AP) at 48 h and 72 h. (**C**) The analysis of percent migration of HPDFs (untreated (UT), treated with 10 ng/mL of TGF-β1, or treated with 10 ng/mL of TGF-β1 with 50 μM of AP) at 0 h, 24 h, 48 h, and 72 h. Figure C(**a**–**d**) present cell migration of the untreated cells at 0 h, 24 h, 48 h, and 72 h, respectively. Figure C(**e**–**h**) present cell migration of TGF-β1-treated cells at 0 h, 24 h, 48 h, and 72 h, respectively. Figure C(**i**–**l**) present cell migration of cells treated with TGF-β1 and 50 μM of AP at 0 h, 24 h, 48 h, and 72 h, respectively. (**D**) Images of scratch wound healing assay in HPDFs (untreated, TGF-β1-treated, or TGF-β1-treated with the presence of 50 μM of AP) at 48 h and 72 h. (**E**) Detection of proliferative marker, Ki-67 (red), in the nuclei of HPDFs (untreated, TGF-β1-treated, or TGF-β1-treated with the presence of 50 μM of AP) at 48 h and 72 h. The cytoplasmic compartment of the cells was elicited by visualizing microtubule (green) stained with an anti-tubulin antibody, and nuclei (blue) were stained with DAPI. Figure E(**a**–**d**) present the untreated cells showing Ki-67 staining, tubulin staining, DAPI staining, and merged images, respectively. Figure E(**e**–**h**) present TGF-β1-treated cells showing Ki-67 staining, tubulin staining, DAPI staining, and merged images, respectively. Figure E(**i**–**l**) present cells treated with TGF-β1 and 50 μM of AP showing Ki-67 staining, tubulin staining, DAPI staining, and merged images, respectively. Scale bar = 500. Data are averages from three independent experiments; * *p* < 0.05 compared to the TGF-β1-treated HPDFs.

To test this hypothesis, we treated HPDFs (seeded at low numbers of cells) with TGF-β1 and AP and observed their cell density over 72 h by a phase-contrast microscope. As presented in Figure 2A(e–h), 10 ng/mL TGF-β1 progressively increased cell density over time compared to the untreated cells (Figure 2A(a–d)); nevertheless, AP drastically suppressed this effect of TGF-β1 on enhancing cell density (Figure 2A(i–l)). The microscopic observation was verified by results from direct cell counting, where cell numbers at 48 and 72 h of the untreated (UT) HPDFs cultured in the serum-free media were 1.21 × 10^5^ ± 1.33 × 10^4^ cells/mL and 2.40 × 10^5^ ± 9.73 × 10^3^ cells/mL, respectively (Figure 2B). However, at these two time points TGF-β1 (10 ng/mL) significantly increased the number of HPDFs to 1.72 × 10^5^ ± 2.19 × 10^4^ cells/mL and 3.33 × 10^5^ ± 2.45 × 10^4^ cells/mL, respectively (Figure 2B). As expected, with the presence of AP, number of TGF-β1-stimulated HPDFs at 48 and 72 h were suppressed to 1.09 × 10^5^ ± 9.60 × 10^3^ cells/mL and 1.71 × 10^5^ ± 1.97 × 10^4^ cells/mL, respectively (Figure 2B). Data from cell migration assay also confirmed that cell migration of TGF-β1-stimulated HPDFs over the course of 72 h was accelerated (Figure 2C(e–h)) compared to that of the untreated HPDFs (Figure 2C (a–d)). As expected, AP could significantly reduce percent migration of TGF-β1-stimulated HPDFs (Figure 2C (i–l)); especially at 48 h and 72 h (Figure 2D). Moreover, to support our findings, we performed an immunofluorescence study to examine the expression level and localization of the Ki-67 protein, because this protein is associated with cell cycle progression and highly expressed in cells that are in the proliferative phase. Results clearly showed that HPDF cells treated with TGF-β1 exhibited high expression of the Ki-67 protein in their nuclei (Figure 2E(e–h)), in comparison to the basal level in the nuclei of the untreated cells (Figure 2E(a–d)). Undoubtedly, the nuclear expression of the Ki-67 protein in TGF-β1-treated HPDFs was drastically reduced when AP was present (Figure 2E(i–l)). These results indicate that AP possesses anti-proliferative effects against the influence of TGF-β1.

### 3.2. Alpinetin Alters Cell Morphological Changes in Human Dermal Fibroblasts Induced by TGF-β1

**Figure 3:**
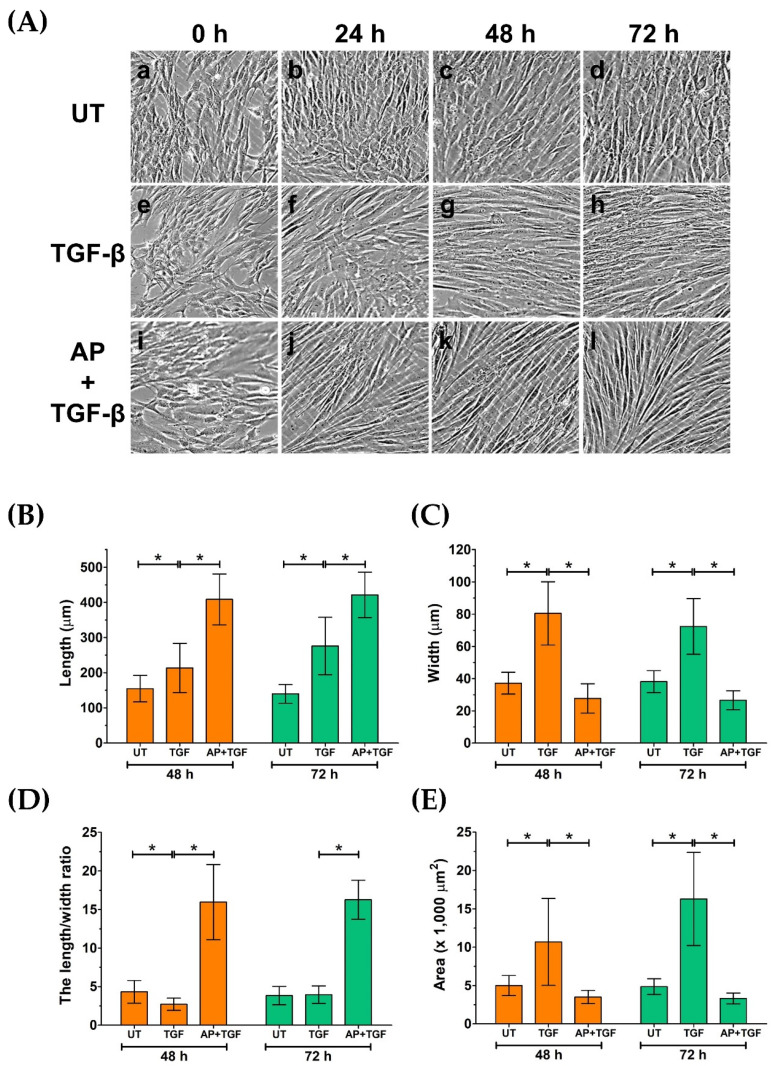
AP inhibits morphological changes in HPDFs from fibroblast to myofibroblast-like structure in response to TGF-β1 stimulation. (**A**) Morphological changes in HPDFs (untreated, TGF-β1-treated, or TGF-β1-treated with the presence of 50 μM of AP) observed by a phase-contrast microscope at 0 h, 24 h, 48 h, and 72 h. Figure A(**a**–**d**) present the untreated cells at 0 h, 24 h, 48 h, and 72 h, respectively. Figure A(**e**–**h**) present TGF-β1-treated cells at 0 h, 24 h, 48 h, and 72 h, respectively. Figure A(**i**–**l**) present cells treated with TGF-β1 and 50 μM of AP at 0 h, 24 h, 48 h, and 72 h, respectively. Quantitative analysis of cell length (**B**), cell width (**C**), the length/width ratio (**D**), and area of HPDFs (**E**). Data are averages from three independent experiments; * *p* < 0.05 compared to the TGF-β1-treated HPDFs.

In addition to an aspect of cell proliferation, we noticed considerable differences in morphology, size, and shape of HPDFs in response to different treatment conditions. Visualization of fibroblast monolayer cells by a phase-contrast microscope showed that at the beginning of treatment (0 h), all groups of HPDFs showed a flat and spindle-like morphology, and these appearances were seen to be unchanged in the untreated group at 24, 48, and 72 h (Figure 3A(a–d)). In contrast, over the course of 72 h post treatment, the size and shape of TGF-β1-treated HPDFs dramatically changed. Specifically, the size of these fibroblasts was larger, and their shape was seen to be likely irregular, with long cytoplasmic extensions; moreover, their nuclei at the cell center could clearly be indicated (Figure 3A(e–h),B–E). Interestingly, HPDFs treated with TGF-β1 with the presence of AP exhibited distinct features which resemble fibrocyte-like morphology (Figure 3A(i–l),B–E). In particular, the individual cells demonstrated a symmetrical elongated and spindle-like morphology which was visibly seen after 24 h. Structurally, these cells appeared to be thinner with longer projections and less cytoplasm, and their nuclei could barely be visualized by a phase-contrast microscope in comparison to TGF-β1-treated fibroblasts (Figure 3A(i–l),B–E).

### 3.3. Alpinetin Inhibits TGF-β1-Induced Formation of Stress Fibers in Human Dermal Fibroblasts

**Figure 4:**
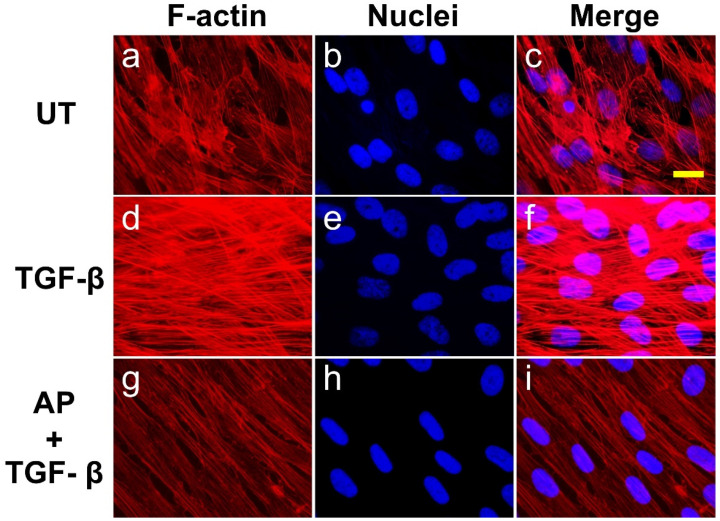
AP reverses the action of TGF-β1 on the formation and organization of actin stress fiber in HPDFs at high cell density. Detection of filamentous actin (F-actin) (red) by using DyLight TM 594-Phalloidin in HPDFs (untreated, TGF-β1-treated, or TGF-β1-treated with the presence of 50 μM of AP) at 48 h. Nuclei of cells (blue) were counterstained with DAPI. Figure (**a**–**c**) present the untreated cells showing F-actin staining, DAPI staining, and merged images, respectively. Figure (**d**–**f**) present TGF-β1-treated cells showing F-actin staining, DAPI staining, and merged images, respectively. Figure (**g**–**i**) present cells treated with TGF-β1 and 50 μM of AP showing F-actin staining, DAPI staining, and merged images, respectively. Observation was conducted at 40× magnification. Scale bar = 500 µm.

On the basis that actin stress fibers are responsible for cell morphological changes, we monitored the expression and organization of filamentous actin using DyLight ^TM^ 594-Phalloidin and the appearance of the nuclei using DAPI. We observed that HPDFs exposed to different treatments demonstrated diverse types of assembly and structure of actin cytoskeleton (Figure 4). After 72 h, HPDFs cultured in media without any treatment demonstrated moderate levels of short and thin actin fiber formation with decent numbers of overlapping areas (Figure 4a–c). Treatment of the fibroblasts with TGF-β1 for 72 h resulted in a tremendous development of thick and elongated actin filaments with distinct and dramatic reorganization. Specifically, TGF-β1 treatment created dense arrays of stress fibers, and these cross-linked networks of filamentous actin were distributed in different layers (Figure 4d–f). The nuclei of TGF-β1-treated HPDFs were oval and aligned randomly in different directions (Figure 4e,f). Interestingly, TGF-β1-treated HPDFs with the presence of AP over 72 h demonstrated less prominent development of stress fibers. The bundles of actin filaments were noticeably elongated, and the cross-linked networks of filamentous actin in different layers disappeared, causing cells to be in a stretching form (Figure 4g–i). Concomitantly, the nuclei of these cells were elongated oval, and their orientation was likely unidirectional (Figure 4h,i).

**Figure 5:**
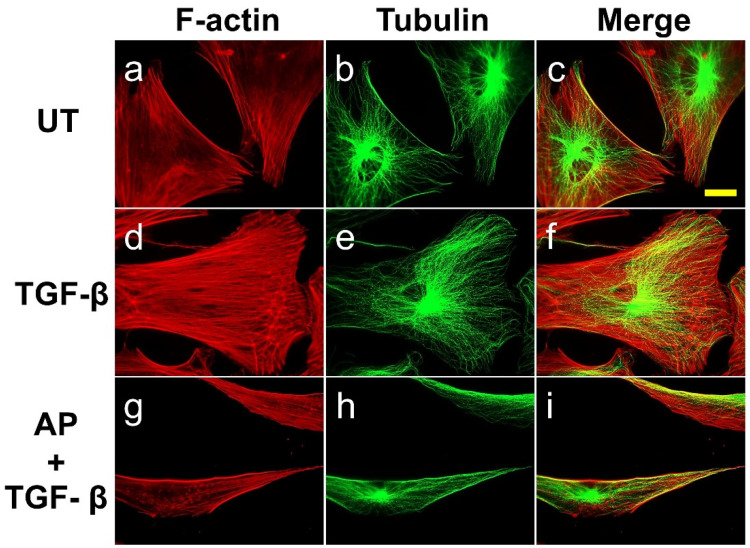
AP blocks the TGF-β1-induced organization of stress fiber and microtubule in individual HPDFs at low cell density. Detection of filamentous actin (F-actin) (red) by using DyLight TM 594-Phalloidin and microtubule (green) by using anti-tubulin antibody in HPDFs (untreated, TGF-β1-treated, or TGF-β1-treated with the presence of 50 μM of AP) at 48 h. Figure (**a**–**c**) present the untreated cells showing F-actin staining, tubulin staining, and merged images, respectively. Picture (**d**–**f**) present TGF-β1-treated cells showing F-actin staining, tubulin staining, and merged images, respectively. Figure (**g**–**i**) present cells treated with TGF-β1 and 50 μM of AP showing F-actin staining, tubulin staining, and merged images, respectively. Observation was conducted at 40× magnification. Scale bar = 500 µm.

To profoundly examine the organization of cytoskeletal protein rearrangements in single cells, we co-stained F-actin together with tubulin in individual HPDFs at low cell density and observed changes in cytoskeletal reorganization. The untreated cells had a rounded-shape morphology and often short protrusions, with characteristic sharp cell edges, extending from the central cell body where the diffused signal of actin was witnessed. The protrusions were likely their focal adhesions, where scattered bundles of actin filaments organized in stress fibers were slightly visible (Figure 5a). Noticeably, thick, sharp, and straight lines of actin filaments accumulated along the cell edges. As detected with the anti-β-tubulin antibody, these untreated dermal fibroblasts exhibited regular microtubule networks emanating from the cell center towards the cell cortex (Figure 5b). The unique arrangement of microtubule filaments at the cell edges co-localized with filamentous actin, creating sharp cellular margins (Figure 5c). Stimulation of HPDFs with 10 ng/mL TGF-β1 for 72 h caused a drastic morphological change concomitant with a robust reorganization of the actin cytoskeleton. TGF-β1-treated cells became larger, flattened out, and well-spread, with fully developed stress fibers throughout the entire cell structure as well as cortical bundles of actin filaments created at the protruding lamellipodia (Figure 5d).

Much like the untreated cells, microtubules in TGF-β1-treated cells appeared intact, emanating from the cell center, and they seemed to be adapted to the changes in cell volume. However, minor restructure of the filaments was observed at the veil-like protrusions, suggesting their migratory organization in coordination with actin filaments (Figure 4e,f). Nevertheless, the presence of AP over the course of TGF-β1 treatment inhibited the formation of these cellular skeletal structures. In particular, AP altered TGF-β1-treated cells to be elongated at both poles, exhibiting a symmetrical cone shape (Figure 5g–i). The abundance of actin stress fibers was reduced (especially at the cell center), but sparse bundles of stress fibers were detected at both elongated poles. Remarkably, AP effectively inhibited the TGF-β1-stimulated formation of lamellipodia. Moreover, one characteristic similar to that of the untreated cells was the formation of thick, sharp, and straight lines of actin filaments and microtubules along the cell edges (Figure 5i).

**Figure 6:**
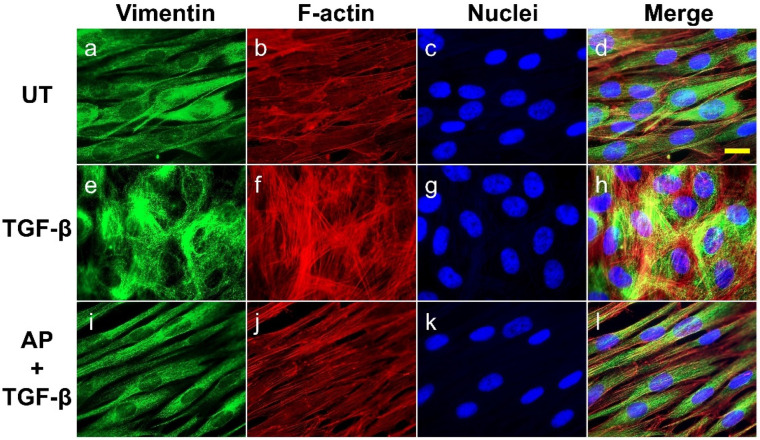
AP reduces the abundance and inhibits the organization of vimentin intermediate filaments upon TGF-β1 induction. HPDFs (untreated, TGF-β1-treated, or TGF-β1-treated with the presence of 50 μM of AP) at 48 h were stained for vimentin (green), F-actin (red), and nuclei (blue). Figure (**a**–**d**) present the untreated cells showing vimentin staining, F-actin staining, DAPI staining, and merged images, respectively. Figure (**e**–**h**) present TGF-β1-treated cells showing vimentin staining, F-actin staining, DAPI staining, and merged images, respectively. Figure (**i**–**l**) present cells treated with TGF-β1 and 50 μM of AP showing vimentin staining, F-actin staining, DAPI staining, and merged images, respectively. Observation was conducted at 40× magnification. Scale bar = 500 µm.

We further determined the abundance and cellular organization of vimentin-positive intermediate filaments, which are distinct mesenchymal features of myofibroblasts. We found that the untreated dermal fibroblast expressed a decent level of vimentin, and the cytoplasmic organization of the filament was seen as a diffused signal along the spindle shape of the cells demonstrating classical vimentin filament structures (Figure 6a–d). A strong increase in signal intensity indicating the upregulation of vimentin was detected in TGF-β1-stimulated cells, and a unique intermediate filament organization was observed. Specifically, TGF-β1-stimulated dermal fibroblasts exhibited dense vimentin filaments which were well-organized in a circular fashion around the nuclei of the cells (Figure 6e–h). Apparently, these vimentin networks were in line with prominently thick actin filament mesh works (Figure 6f–h). However, AP treatment completely blocked the effects of TGF-β1 on vimentin cytoskeletal alterations in dermal fibroblasts. These fibroblasts showed a diffused vimentin signal in the cytoplasm along the axis of the cell body, which was discrete, elongated, and aligned with fewer and thinner filamentous actin (Figure 6i–l).

### 3.4. Alpinetin Reduces the Effects of TGF-β1 on Inducing β-Catenin and N-Cadherin Expression in Human Dermal Fibroblasts

**Figure 7:**
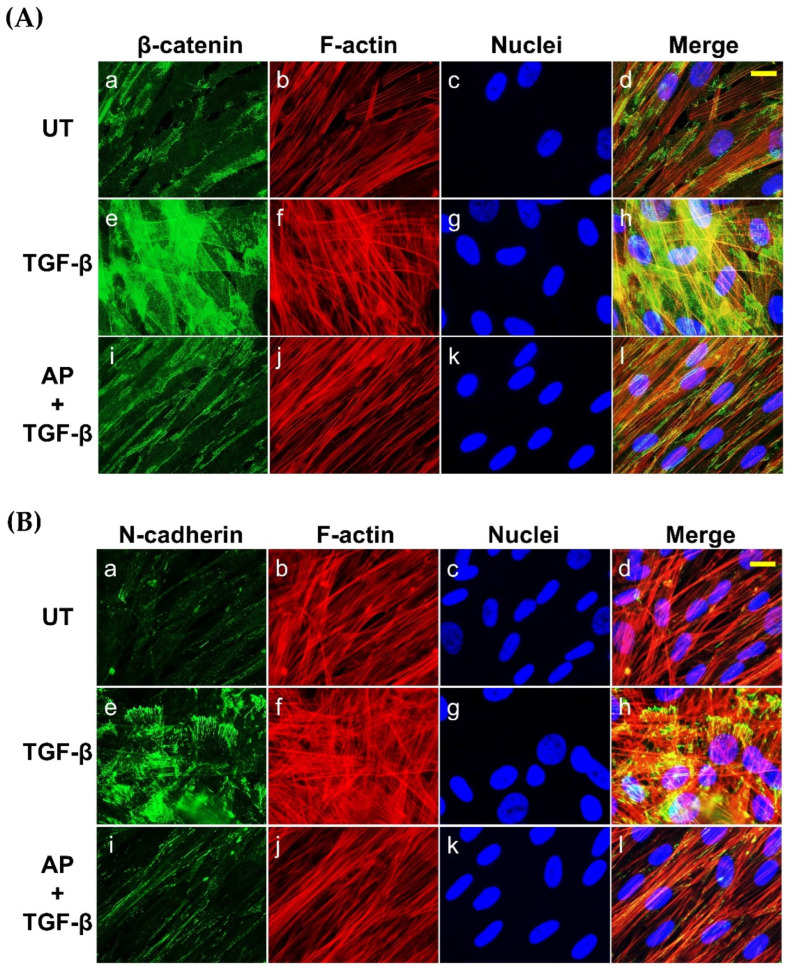
AP suppresses the synthesis and inhibits the organization of β-catenin and N-cadherin in response to TGF-β1 induction. (**A**) HPDFs (untreated, TGF-β1-treated, or TGF-β1-treated with the presence of 50 μM of AP) at 48 h were stained for β-catenin (green), F-actin (red), and nuclei (blue). (**B**) HPDFs (untreated, TGF-β1-treated, or TGF-β1-treated with the presence of 50 μM of AP) at 48 h were stained for N-cadherin (green), F-actin (red), and nuclei (blue). Figure A(**a**–**d**) present the untreated cells showing β-catenin staining, F-actin staining, DAPI staining, and merged images, respectively. Figure A(**e**–**h**) present TGF-β1-treated cells showing β-catenin staining, F-actin staining, DAPI staining, and merged images, respectively. Figure A(**i**–**l**) present cells treated with TGF-β1 and 50 μM of AP showing β-catenin staining, F-actin staining, DAPI staining, and merged images, respectively. Figure B(**a**–**d**) present the untreated cells showing N-cadherin staining, F-actin staining, DAPI staining, and merged images, respectively. Figure B(**e**–**h**) present TGF-β1-treated cells showing N-cadherin staining, F-actin staining, DAPI staining, and merged images, respectively. Figure B(**i**–**l**) present cells treated with TGF-β1 and 50 μM of AP showing N-cadherin staining, F-actin staining, DAPI staining, and merged images, respectively. Observation was conducted at 40 X magnification. Scale bar = 500 µm.

**Figure 8:**
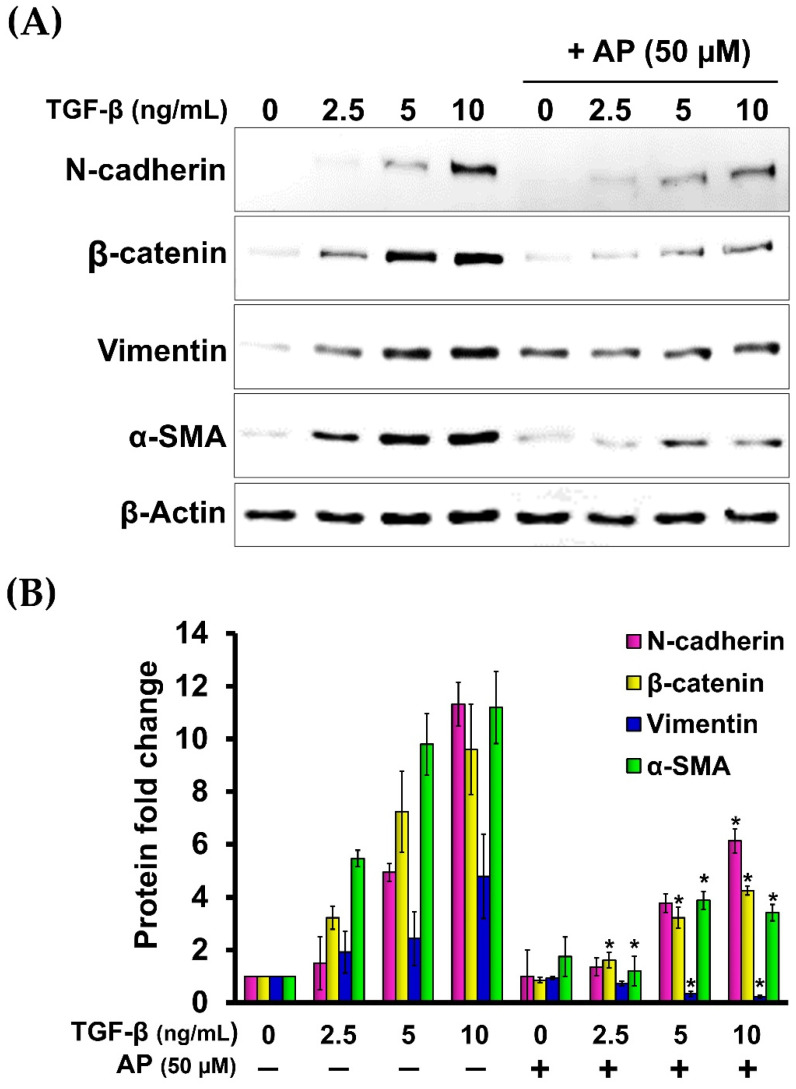
AP potently decreases the expression of key downstream fibrotic hallmarks of TGF-β1. (**A**) Western blot analysis of HPDFs (untreated, TGF-β1-treated, or TGF-β1-treated with the presence of 50 μM of AP) at 48 h showing a dose-dependent decrease in the TGF-β1-induced production of N-cadherin, β-catenin, vimentin, and α-SMA. β-Actin was used as a loading control and for normalization. (**B**) Densitometric analysis of the band intensity of each protein in HPDFs (untreated, TGF-β1-treated, or TGF-β1-treated with the presence of 50 μM of AP) at 48 h; * *p* < 0.05 compared to TGF-β1-treated HPDFs.

Since it has been demonstrated that TGF-β1 potently induces the expression of β-catenin and N-cadherin in fibroblasts from many tissues, we then evaluated the effects of AP on reducing the production of these two proteins. As presented in Figure 7, the untreated cells cultured in serum-free media showed low basal level of β-catenin and N-cadherin. Upon TGF-β1 treatment, the expression of β-catenin and N-cadherin was increased when the concentration of TGF-β1 was increased. TGF-β1 at the highest concentration (10 ng/mL) maximally upregulated the expression of β-catenin and N-cadherin to approximately 9-fold and 11-fold, respectively (Figure 8A,B). Dermal fibroblasts stimulated with TGF-β1 with the presence of AP at 12.5, 25, and 50 μM resulted in a significant decrease in β-catenin and N-cadherin signal intensity and inhibition of their organization (Figure 8A,B). Moreover, we confirmed our Western blot data with immunofluorescence and visualized that the intensity of β-catenin and N-cadherin in the untreated cells was weak, with their main localization at the cell–cell contact (Figure 7A(a–d),B(a–d)). In contrast, TGF-β1 strongly enhanced the positive signal of β-catenin and N-cadherin in the cells, especially at the areas of cell–cell interaction where they exhibited special organization patterns (Figure 7A(e–h),B(e–h)). Remarkably, AP treatment could effectively reduce the intracellular signal, and the staining patterns of β-catenin and N-cadherin were relatively similar to those of the untreated fibroblasts (Figure 7A(i–l),B(i–l)). Consistent with these findings, we observed that AP could greatly repress the TGF-β1-induced expression of α-SMA and vimentin, which are crucial markers of dermal fibrosis (Figure 8A,B).

### 3.5. Alpinetin Downregulates the Expression of α-Smooth Muscle Actin (α-SMA) and Type I Collagen Component (Col1A1) in Human Dermal Fibroblasts Stimulated with TGF-β1

**Figure 9:**
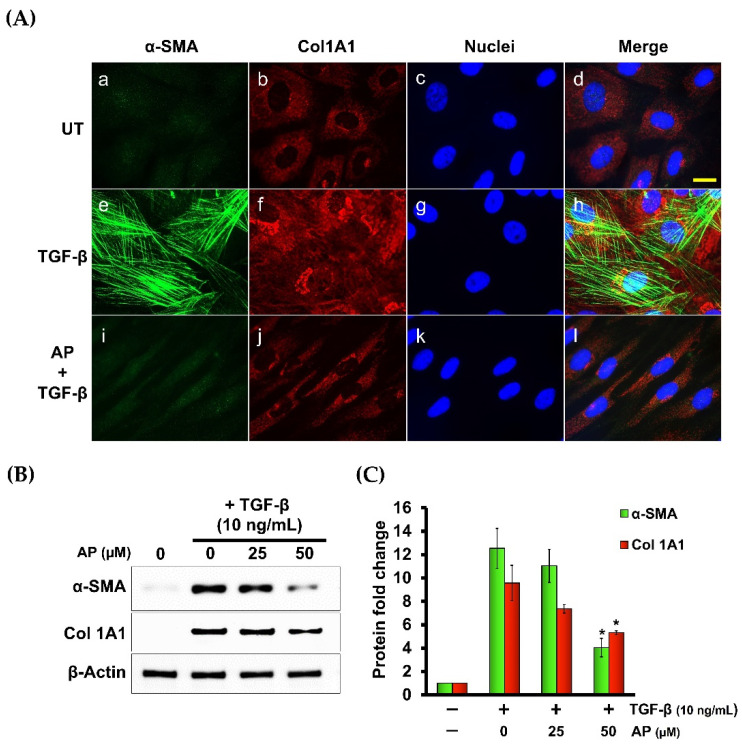
AP reduces the TGF-β1-stimulated expression of α-SMA and component of type I collagen (Col1A1). (**A**) Immunofluorescence staining of α-SMA (green) and Col1A1 (red) of HPDFs (untreated, TGF-β1-treated, or TGF-β1-treated with the presence of 50 μM of AP) at 48 h. Nuclei were counterstained with DAPI (blue). Figure A(**a**–**d**) present the untreated cells showing α-SMA staining, Col1A1staining, DAPI staining, and merged images, respectively. Figure A(**e**–**h**) present TGF-β1-treated cells showing α-SMA staining, Col1A1staining, DAPI staining, and merged images, respectively. Figure A(**i**–**l**) present cells treated with TGF-β1 and 50 μM of AP showing α-SMA staining, Col1A1staining, DAPI staining, and merged images, respectively. (**B**) Western blot analysis showing dose-dependent effects of AP on reducing the TGF-β1-induced expression of α-SMA and Col1A1. β-Actin was used as a loading control and for normalization. (**C**) Quantitative analysis of the Western blot band intensity of α-SMA and Col1A1 of HPDFs (untreated, TGF-β1-treated, or TGF-β1-treated with the presence of 25 and 50 μM of AP) at 48 h. Scale bar = 500 µm. * *p* < 0.05 compared to TGF-β1-treated HPDFs.

Since we observed that AP inhibited TGF-β1-induced formation of stress fibers in HPDFs, we hypothesized that AP may be able to block the potent effects of TGF-β1 on inducing the expression of α-SMA and type I collagen (Col1A1). Data from immunofluorescence study revealed that TGF-β1 strongly induced the expression of α-SMA, with a unique lattice characteristic (Figure 9A(e–h)) in comparison to the negative expression of the untreated cells (Figure 9A(a–d). As expected, AP completely suppressed α-SMA expression in response to TGF-β1 stimulation, thus α-SMA lattice formation was not visualized (Figure 9A(i–l)). In accordance with this observation, we noticed that compared with the untreated fibroblasts which expressed a low basal level of induced type 1 collagen (Figure 9A(b–d)), cells stimulated with TGF-β1 exhibited higher expression of type 1 collagen (Col1A1) (Figure 9A(f–h)). However, AP effectively reduced TGF-β1-induced type 1 collagen (Figure 9A(j–l)).

## 4. Discussion

Fibrosis is a consequence of a complex pathological scarring process associated with overproduction of extracellular matrix (ECM), dysregulation of ECM turnover, and contribution of high tissue cellularity. Like in other tissues, skin fibrosis is caused by ECM deposition, but this dermatologic disease is also rooted in excessive proliferation of dermal fibroblasts [3]. It is known that skin injuries lead to skin fibrotic diseases such as keloids, resulting from excessive deposition of ECM in the dermis and subcutaneous tissues [3]. With this reason, treating skin fibrosis is practically challenging. It is well characterized that dermal fibroblasts constitutively activated by TGF-β contribute to skin fibrosis [5,6]. Therefore, agents or compounds that target the overactivation of fibroblasts would elicit potential anti-fibrotic effects.

Here, we utilized dermal fibroblasts (the primary effector cell in skin fibrosis) as a pre-clinical model for defining the effect of a potential agent, alpinetin (AP). Since the effects of TGF-β on fibroblast proliferation have been reported [30,31,32], we examined the potential effects of AP on the proliferative status of human primary dermal fibroblasts (HPDFs) and found that AP could strongly inhibit the metabolic activity and cell number of HPDFs stimulated with TGF-β. We deeply explored that AP was effective in reducing the expression of the Ki-67 protein in the nuclei of TGF-β-stimulated HPDFs. This protein is well characterized as an important marker for cell proliferation [33,34]. These results indicate that AP possesses anti-proliferative effects against the influence of TGF-β. Consistent with these results, AP exhibited inhibitory effects on TGF-β-induced migration of dermal fibroblasts, and this may possibly be a consequence of reduced cell proliferation. Interestingly, by observing the cells under a phase-contrast microscope with higher magnification, we noticed that AP strongly reversed TGF-β-induced morphological alterations, and in addition exhibited a symmetrical elongated and spindle-like morphology. On the basis that TGF-β treatment affects the morphology of several different cell types [35,36,37], in part through stimulating the assembly of actin filaments into stress fibers [37], we monitored the interfering effects of AP on TGF-β-induced formation of actin stress fiber. As hypothesized, AP potently disrupted the abundance of actin stress fibers and the rearrangement of actin–tubulin cytoskeletal components required for the formation of lamellipodia. These results support our earlier findings that AP delayed dermal fibroblast migration. In addition, TGF-β possesses its fibrogenic actions by inducing phenoconversion from fibroblasts to myofibroblasts. One of the distinct molecular hallmarks contributing to mesenchymal features is the upregulation of vimentin intermediate filaments [10,38]. We discovered that AP could effectively inhibit the TGF-β-stimulated expression and reorganization of vimentin in primary dermal fibroblasts, and these data convince that AP can function against the fibrogenic actions of TGF-β on myofibroblast phenoconversion.

Although the canonical TGF-β pathway has been identified as a major factor responsible for the pro-fibrotic signaling cascade sufficient for inducing fibrosis [39], there is accumulating evidence revealing the molecular interdependence between this signaling and others, especially the Wnt/β-catenin signaling pathway [19]. It has been demonstrated that TGF-β upregulated β-catenin expression in human lung fibroblasts [16]. Expression of β-catenin in the animal dermis caused spontaneous skin fibrosis with elevated fibroblast proliferation [40,41,42]. Particularly, Wnt/β-catenin signaling induced fibroblast proliferation and motility [43,44]. Our current study supports previous studies, since we also observed that the expression of β-catenin was increased in response to increased concentrations of TGF-β with the concomitance of an increase in fibroblast proliferation. Intriguingly, AP strongly suppressed the influence of TGF-β on driving β-catenin expression. Thus, a possible explanation of the anti-proliferative effect of AP on TGF-β-induced human dermal fibroblasts may be from its ability to block β-catenin expression. In addition to the well-characterized functions of β-catenin in dermal fibroblast proliferation, sustained expression of β-catenin in dermal fibroblasts could upregulate the expression of specific extracellular matrix (ECM)-encoding genes, resulting in altered morphology and skin fibrosis [20]. Since AP strongly blocked the impact of TGF-β on inducing dermal fibroblast morphological changes, one possible mechanism of AP on this event may be rooted in the potency of this compound on suppressing β-catenin expression and the production of β-catenin-induced fibrotic ECM.

Furthermore, it has been demonstrated that the N-cadherin prodomain at the cell surface is a potential biomarker of pathological myofibroblasts and fibrosis in many tissues [24], and that TGF-β derived from colon cancer cells could induce N-cadherin expression, which consequently promoted invasion of myofibroblasts [23]. In association with cell motility and invasion, N-cadherin molecules were complexed with β-actin and the α-SMA cytoskeleton, and the findings suggested that N-cadherin might stimulate invasion by forming adherens junctions linked to the cytoskeleton [45]. Therefore, data from our current study showing that AP effectively reduced TGF-β-induced N-cadherin expression in human dermal fibroblasts are suggestive that the inhibitory effects of AP on myofibroblast migration may partly be due to its ability to downregulate N-cadherin expression. We further determined the effects of AP on the TGF-β-stimulated expression of α-SMA cytoskeleton and discovered that AP strongly suppressed α-SMA expression even when the concentration of TGF-β was increased. Consistent with this result, AP could also reduce the expression of type I collagen, which has been reported to exhibit higher expression upon TGF-β stimulation [13]. Overproduction of collagens has been associated with pathogenesis of skin fibrosis. For instance, biomarkers of type I, III, and VI collagen formation were reported to be elevated in patients with systemic sclerosis (SSc), and several growth factors and cytokines including TGF-β play a major role in stimulating collagen synthesis [46,47]. Likewise, excessive deposition of extracellular matrix such as collagen in the dermis and subcutaneous tissues has proved to be a key cause of keloids [48]. We disclosed that AP reduced the deposition of type I collagen, and this suggests that AP may possess potential anti-fibrotic activities, at least in part through its capability to block the biosynthesis and accumulation of collagen and other ECMs.

It has been shown that PPARγ abrogated TGF-β-induced stimulation of collagen gene expression, myofibroblast transdifferentiation, and Smad-dependent promoter activity in normal fibroblasts [49]. Specifically for human dermal fibroblasts, it was revealed that PPARγ agonists inhibited TGF-β1 and matrix production [50,51]. Moreover, the potent effects of PPARγ agonists on inhibiting pro-fibrotic phenotypes of fibroblasts in other tissues have been reported [52,53]. Therefore, it is reasonable that the possible anti-fibrotic mechanism of AP may be due to its ability to activate PPARγ, since AP is a natural plant-derived flavonoid characterized to be a PPARγ agonist exhibiting anti-inflammatory effects [54,55].

Current advanced treatments of skin conditions associated with aberrant TGF-β signaling are available, but certain kinds of skin fibrotic diseases such as keloids are difficult to treat, and they easily relapse [4]. Our discovery showing that a natural PPARγ agonist, alpinetin, could slow the rate of proliferation, block the biosynthesis of collagen components, and inhibit the formation of actin stress fiber and the expression of vimentin, α-SMA, β-catenin, and N-cadherin, provides a possibility to develop it as a novel anti-fibrotic agent with a distinct mechanism of action. This may be beneficial for patients with skin fibrosis who are irresponsive to currently available therapeutic strategies. However, the current study has limitations in certain aspects, including the use of human primary dermal fibroblasts originated from one donor. To ensure the conserved properties of AP, future studies using dermal fibroblasts isolated from multiple sources including normal and keloid tissue samples (as well as animal studies and human clinical trials) would provide more precise data beneficial for encouraging clinical significance of this natural compound.

## 5. Conclusions

The current study discovered that the natural compound alpinetin could effectively block the fibrotic effects of TGF-β1 in human primary dermal fibroblasts. Alpinetin reversed many different key cellular hallmarks responsible for the pathogenesis of skin fibrosis. The possible mechanisms of alpinetin may be associated with its pharmacological property as a PPARγ agonist. However, this aspect needs to be further elucidated. Our in vitro study provided convincing data suggesting the possible anti-fibrotic effects of alpinetin. Further investigation in animal models and clinical studies would verify its safety and efficacy and ensure that this active compound can be developed as an alternative agent for skin fibrosis treatment.

## Data Availability

All data, tables, and figures are original and are available in this article.
